# Outcomes of HIV-infected versus HIV-non-infected patients treated for drug-resistance tuberculosis: Multicenter cohort study

**DOI:** 10.1371/journal.pone.0193491

**Published:** 2018-03-08

**Authors:** Mathieu Bastard, Elisabeth Sanchez-Padilla, Philipp du Cros, Atadjan Karimovich Khamraev, Nargiza Parpieva, Mirzagaleg Tillyashaykov, Armen Hayrapetyan, Kamene Kimenye, Shazina Khurkhumal, Themba Dlamini, Santiago Fadul Perez, Alex Telnov, Cathy Hewison, Francis Varaine, Maryline Bonnet

**Affiliations:** 1 Epicentre, Paris, France; 2 Médecins Sans Frontières, London, United Kingdom; 3 Teaching Assistant of the Department of Public Health Administration, Nukus branch of Tashkent Pediatric Medical Institute, Nukus, Uzbekistan; 4 National Tuberculosis Institute, Tashkent, Uzbekistan; 5 National Tuberculosis Control Office, Erevan, Armenia; 6 Programmatic Management of Drug resistant Tuberculosis, Nairobi, Kenya; 7 National Tuberculosis Program, Tbilisi, Georgia; 8 MoH-TB National Control Program National Manager, Mbanane, Swaziland; 9 National Public Health Institute, Respiratory disease department, Bogota, Colombia; 10 Médecins Sans Frontières, Geneva, Switzerland; 11 Médecins Sans Frontières, Paris, France; 12 Unité Mixte Internationale UMI233-U1175, Institute of Research for Development, Montpelier, France; Michigan State University College of Veterinary Medicine, UNITED STATES

## Abstract

**Background:**

The emergence of resistance to anti-tuberculosis (DR-TB) drugs and the HIV epidemic represent a serious threat for reducing the global burden of TB. Although data on HIV-negative DR-TB treatment outcomes are well published, few data on DR-TB outcomes among HIV co-infected people is available despite the great public health importance.

**Methods:**

We retrospectively reported and compared the DR-TB treatment outcomes of HIV-positive and HIV-negative patients treated with an individualized regimen based on WHO guidelines in seven countries: Abkhazia, Armenia, Colombia, Kenya, Kyrgyzstan, Swaziland and Uzbekistan.

**Results:**

Of the 1,369 patients started DRTB treatment, 809 (59.1%) were multi-drug resistant (MDR-TB) and 418 (30.5%) were HIV-positive. HIV-positive patients were mainly from African countries (90.1%) while HIV-negative originated from Former Soviet Union (FSU) countries. Despite a higher case fatality rate (19.0% vs 9.4%), HIV-positive MDR-TB patients had a 10% higher success rate than HIV-negative patients (64.0% vs 53.2%, p = 0.007). No difference in treatment success was found among polydrug-resistant (PDR-TB) patients. Overall, lost to follow-up rate was much higher among HIV-negative (22.0% vs. 8.4%). Older age and not receiving ART were the only factors associated with unfavorable treatment outcome among HIV-positive patients.

**Conclusions:**

As already known for HIV-negative patients, success rate of DR-TB HIV-positive patients remains low and requires more effective DR-TB regimen using new drugs also suitable to HIV-infected patients on ART. The study also confirms the need of ART introduction in HIV co-infected patients.

## Introduction

Drug-resistant TB (DR-TB), especially multidrug-resistance TB (MDR-TB) is a major public health concern and an obstacle to control of TB over the world. There were an estimated 600 000 (range, 540 000–660 000) incident cases of MDR/RR-TB in 2016, with cases of MDR-TB accounting for 82% (490 000) of the total and MDR-TB represented 4.1% (95%CI 2.8–5.3%) of new TB cases and 19.0% (95%CI 9.8–27.0%) of previously treated cases. In the same year, 57% of notified TB patients had a documented HIV test result. In the WHO African Region, where the burden of HIV-associated TB is highest, 82% of TB patients had a documented HIV test result. A total of 476,774 TB cases among HIV-positive people were reported and of these, 85% were on antiretroviral therapy (ART). [1,2]. Although DR-TB prevalence among HIV-infected patients is still not clearly documented, recent studies in high burden African regions show alarming levels of DR-TB [3,4]. In addition, a recent meta-analysis of twenty four pooled studies has shown that the odds of having MDR-TB among HIV-positive patients is significantly higher by 24% [5]. This trend is also revealed in eastern and central Europe and central and in Swaziland by the Global TB program [6]. Another meta-analysis showed that the success rate among HIV co-infected patients similar to success rates reported among MDR-TB patients in general, regardless of HIV status [7].

DR-TB treatment outcomes are still poor, particularly among MDR-TB with a treatment success rate of only 52% [1]. This proportion tends to be lower among African countries, where HIV prevalence is the highest. However, few data on DR-TB outcomes among HIV co-infected people is available despite the great public health importance. We have conducted a retrospective multicentric analysis of seven DR-TB programs supported by Médecins sans Frontières (MSF) between 2001 and 2015 to describe and compare treatment outcomes among HIV-positive and HIV-negative DR-TB patients and to identify predictors of unsuccessful outcomes among HIV-positive patients.

## Methods

### Study setting

We routinely collected data in DR-TB programs in seven countries: Abkhazia, Armenia, Colombia, Kenya, Kyrgyzstan, Swaziland and Uzbekistan. Four of these countries were considered high MDR-TB burden (Armenia, Georgia, Kyrgyzstan and Uzbekistan) with the proportion of MDR-TB amongst new TB cases ranging from 11.0% in Armenia to 32.0% in Kyrgyzstan, and amongst previously treated TB cases ranging from 33.0% in Georgia to 63.0% in Uzbekistan. The prevalence of HIV among adults ranged from an estimated 0.2% (0.1–0.2) in Uzbekistan to 28.8% (26.7–30.5) in Swaziland (S1 Table) [8].

In these programs, patients received an individualized treatment regimen based on Drug Susceptibility Testing (DST) results. MDR-TB regimens included an injectable drug during the intensive phase (kanamycin or capreomycin), a fluoroquinolone throughout, and other WHO Group 4 second-line drugs (para-aminosalicylic acid, ethionamide or prothionamide, cycloserine) or Group 5 drugs (clofazimine, amoxicillin-clavulanate acid and/or clarithromycin). Patients with Isoniazid (H) resistance were treated with the combination of rifampicin (R), ethambutol (E) and pyrazinamide (Z) for 9 months with the possibility of the addition of a fluoroquinolone in patients with extensive disease. Patients with other polydrug-resistant (PDR) patterns (resistance to more than one first-line anti-TB drug, other than both isoniazid and rifampicin) received a regimen combining first line anti-tuberculosis drugs to which they were still susceptible plus a fluoroquinolone and an injectable during the first months of treatment for a total treatment duration of 12 to 18 months [9,10]. The full course of treatment was administrated under direct observation six days a week and with socioeconomic and psychological support. DRTB treatment was provided either at a health facility or at home by a health care provider to facilitate the adherence to treatment after hospital discharge.

### Study population and definitions

Databases were administratively censored in July 2015 for Abkhazia, Armenia, Colombia, Kenya, Kyrgyzstan and Swaziland, and in August 2013 for Uzbekistan. Patients were included in this study if they had a baseline DST confirming DR-TB, if they had a known HIV status and if they started on DRTB treatment at least 24 months before the closure date of the database to allow for DR-TB outcome assessment.

PDR-TB patients’ resistance profile at baseline were defined as follow: H(S) for patients with resistant strain to isoniazid plus or minus resistance to streptomycin and susceptible to rifampicin; HE(S) for those with resistant strain to isoniazid and ethambutol and susceptible to rifampicin; R(E)(S) for those with resistant strain to rifampicin and susceptible to isoniazid. PDR-TB that could not be classified as one of these three categories were defined as “other PDRTB”. MDR-TB patients’ strain resistance profile at baseline were defined as follows: MDR with DST to second-line drugs not known; simple MDR (no resistance to ofloxacin and injectables); pre-XDR one injectable (resistance to either kanamycin or capreomycin and ofloxacin-susceptible); pre-XDR two injectables (resistance to kanamycin and capreomycin and ofloxacin-susceptible); pre-XDR ofloxacin (resistance to ofloxacin and susceptibility to both kanamycin and capreomycin); and XDR (resistance to ofloxacin and resistance to either kanamycin or capreomycin).

Treatment outcomes followed the 2008 WHO definition [9]: cured—treatment completion and at least five (three for PDR-TB) consecutive negative culture results during the final 12 months (6 months for PDR-TB) of treatment for MDR-TB failure—positive culture results in ≥2 of the five cultures recorded in the final 12 months or in any one of the final three cultures, or if a clinical decision was made to discontinue treatment early; treatment completed—if the definition of cured was not met due to lack of bacteriological results for MDR-TB and as at least one positive culture after 3 months of adapted regimen for PDR-TB; death; and loss to follow up—patient missed ≥2 consecutive months of treatment. Then, we defined a treatment outcome as successful if patient was cured or completed treatment, and unsuccessful if patient died, failed treatment or were lost to follow-up [9].

### Statistical analysis

Patients’ characteristics at treatment initiation were stratified by HIV status and were summarized using frequencies and percentages for categorical variables, and median and interquartile range (IQR) for continuous variables. Treatment outcomes were stratified and compared by baseline resistant profiles and by HIV status. Comparisons were made using Chi-squared test for categorical variables and Wilcoxon rank-sum test for continuous variables.

Unsuccessful outcome rates, mortality and lost to follow-up rates were described using Kaplan-Meier estimates and compared with log-rank tests. Univariate and multivariate Cox proportional-hazards models were fitted to explore risk factors of treatment outcomes of DRTB HIV co-infected patients. The following covariates were included in the univariate analysis: program location, gender, age, being single, being ex-prisoner, being unemployed, contact with a MDR patient, body mass index (BMI), past history of anti-TB treatment, presence of cavities on chest X-ray, diabetes, sputum smear-microscopy result, DST profile at treatment initiation, HIV antiretroviral treatment (ART) and cotrimoxazole prophylaxis. Covariates of clinical interest and those associated with a p-value <0.4 in univariate analysis were included in the initial multivariate model; a manual backward stepwise approach was used to obtain the final multivariate model. Statistical significance (p-value < 0.05) was assessed with the likelihood-ratio test. Proportional-hazards assumption was checked by testing the Schoenfeld residuals. Sensitivity analysis excluding patients who were lost to follow-up was also carried out in order to check if the association between covariates and unfavorable outcome remained unchanged when patients who were lost to follow-up were excluded. Analyses were performed using Stata 13.1 software (Stata Corporation, College Station, Texas, USA).

### Ethical approval

This research fulfilled the exemption criteria set by the Médecins Sans Frontières Ethics Review Board for a posteriori analyses of routinely collected clinical data and thus did not require MSF ERB review. All data were fully anonymized before data entry. It was conducted with permission from each medical director of each Médecins Sans Frontières section.

## Results

### Baseline characteristics of patients

A total 1,369 patients who started DRTB treatment from November 2006 to June 2013 were included in this study. Among them, 418 (30.5%) were positive for HIV and 809 (59.1%) were MDR-TB. The prevalence of HIV was 25.3% (108/426) among PDR-TB patients and 26.1% (211/809) among MDR-TB patients (p = 0.781). HIV-positive patients came mainly from African countries (90.1%) while HIV-negative originated from Former Soviet Union (FSU) countries. There were significantly more females and the median age was lower among HIV infected compared to HIV negative patients. HIV-positive patients were more likely to have been treated with first line antituberculosis drugs whereas HIV negative patients have been more exposed to second line drugs. MDR-TB contact history and presence of lung cavitations were more common among HIV negative patients. Both isoniazid monoresistance and Rifampicin resistance other than MDR-TB were more common among HIV-negative patients. No major differences were shown among MDR-TB patients. (Table 1).

**Table 1:** Characteristics of DRTB patients at treatment start stratified by HIV status.

Characteristics	HIV negative	HIV positive	p-value	Overall
	N = 951	N = 418		N = 1,369
	N(%)	N(%)		N(%)
**Project location**			<0.001	
Abkhazia	17 (1.8)	13 (3.1)		30 (2.2)
Armenia	528 (55.5)	40 (9.6)		568 (41.5)
Colombia	53 (5.6)	1 (0.2		54 (3.9)
Kenya	44 (4.6)	27 (6.5)		71 (5.2)
Kyrgyzstan	93 (9.8)	4 (1.0)		97 (7.1)
Swaziland	80 (8.4)	333 (79.7)		413 (30.2)
Uzbekistan	136 (14.3)	0 (0.0)		136 (9.9)
**Gender**			<0.001	
Male	683 (71.8)	195 (46.6)		878 (64.1)
Female	268 (28.2)	223 (54.4)		491 (35.9)
**Age (years)**			0.001	
Median [IQR]	36 [26–48]	33 [27–40]		34 [27–46]
< 35	445 (47.0)	236 (57.3)		681 (50.1)
≥ 35	501 (53.0)	176 (42.7)		677 (49.9)
Missing	5	6		11
**Ex-prisoner**			0.084	
No	834 (87.7)	380 (90.9)		1,214 (88.7)
Yes	117 (12.3)	38 (9.1)		155 (11.3)
**Contact of a MDR-TB case**			<0.001	
No	835 (87.9)	406 (97.1)		1,241 (90.7)
Yes	115 (12.1)	12 (2.9)		127 (9.3)
Missing	1	0		1
**History of previous anti-TB treatment**			<0.001	
New case	417 (45.1)	106 (25.9)		523 (39.2)
Previously treated with FLD	357 (38.6)	265 (64.6)		622 (46.6)
Previously treated with SLD	130 (14.0)	29 (7.1)		159 (11.9)
Transferred in	21 (2.3)	10 (2.4)		31 (2.3)
Missing	26	8		34
**BMI**			0.201	
< 18.5	205 (32.9)	142 (36.8)		347 (34.4)
≥ 18.5	419 (67.1)	244 (63.2)		663 (65.6)
Missing	327	32		359
**Cavities on CXR**			<0.001	
No	300 (31.5)	312 (74.6)		612 (44.7)
Yes	651 (68.5)	106 (25.4)		757 (55.3)
**Diabetes**			-	
No	746 (91.4)	418 (100)		1,164 (94.3)
Yes	70 (8.6)	0 (0.0)		70 (5.7)
Missing	135	0		135
**Smear result**			0.014	
Negative	272 (32.5)	116 (32.0)		388 (32.4)
1+	183 (21.9)	94 (26.0)		277 (23.1)
2+	158 (18.9)	41 (11.3)		199 (16.6)
3+	223 (26.6)	111 (30.7)		334 (27.9)
Scanty	1 (0.1)	0 (0.0)		1 (0.1)
Missing	114	56		170
**DST profile**†				
**PDR**			<0.001	
H(S)	211 (73.3)	27 (33.3)		238 (64.5)
HE(S)	44 (15.3)	36 (44.5)		80 (21.7)
R(E)(S)	33 (11.5)	18 (22.2)		51 (13.8)
Other	30	27		57
**MDR/XDR**			0.459	
H and R resistant only	211 (59.9)	24 (63.2)		235 (60.3)
Pre-XDR (1 injectable)	33 (9.4)	6 (15.8)		39 (10.0)
Pre-XDR (2 injectables)	36 (10.2)	1 (2.6)		37 (9.5)
Pre-XDR Fluoroquinolones	42 (11.9)	4 (10.5)		46 (11.8)
XDR	30 (8.5)	3 (7.9)		33 (8.5)
SLD line resistance missing	246	173		419

^†^ 134 DRTB patients could not be classified as PDR or MDR/XDR

Cotrimoxazole prophylaxis was prescribed for 97.5% of co-infected patients. A total of 317 (75.8%) co-infected patients were started on ART: 238 (75.1%) in a median time of 7.7 months [IQR 2.8–23.7] before the start of DRTB treatment and 75 (24.9%) in a median time of 1.4 months [IQR 0.7–4.3] after the start of DRTB treatment. The main prescribed ART regimens were: TDF/3TC/EFV (34.7%), AZT/3TC/EFV (28.9%), AZT/3TC/NVP (12.9%) and d4T/3TC/EFV (8.0%).

### Treatment outcomes

Despite a higher case fatality rate (19.0% vs 9.4%), HIV-positive MDR-TB patients had a 10% higher success rate than HIV-negative patients (64.0% vs 53.2%, p = 0.007), mostly explained by the very high lost to follow-up rate of HIV-negative patients (26.2%). In the group of PDR-TB patients, the difference of success rate seems to be more in favor of HIV-negative patients (76.7% vs 68.5%, p = 0.09) due to the large difference of case fatality between HIV-positive and negative patients (19.4% vs 2.8%). The lost to follow-up rates were two times lower among HIV-positive patients compared to HIV-negative both for PDR-TB and MDR-TB patients (Table 2).

**Table 2:** Treatment outcomes of DRTB patients stratified by DST profiles and HIV status.

Treatment outcome	HIV-negative	HIV-positive	p-value	Overall
**PDR-TB patients (N = 426)**			<0.001	
Cured	148 (46.5)	55 (50.9)		203 (47.6)
Completed treatment	96 (30.2)	19 (17.6)		115 (27.0)
*Success*	*244 (76*.*7)*	*74 (68*.*5)*		*318 (74*.*6)*
Died	9 (2.8)	21 (19.4)		30 (7.0)
Failed	19 (6.0)	7 (6.5)		26 (6.1)
Lost to follow-up	46 (14.5)	6 (5.6)		52 (12.2)
**MDR-TB patients (N = 809)**			<0.001	
Cured	240 (40.1)	102 (48.3)		342 (42.3)
Completed treatment	78 (13.0)	33 (15.6)		111 (13.7)
*Success*	*318 (53*.*2)*	*135 (64*.*0)*		*453 (56*.*0)*
Died	56 (9.4)	40 (19.0)		96 (11.9)
Failed	67 (11.2)	11 (5.2)		78 (9.6)
Lost to follow-up	157 (26.2)	25 (11.8)		182 (22.5)
**Overall (N = 1,369)**			<0.001	
Cured	413 (43.4)	221 (52.9)		634 (46.3)
Completed treatment	175 (18.4)	63 (15.1)		238 (17.4)
*Success*	*588 (61*.*8)*	*284 (67*.*9)*		*872 (63*.*7)*
Died	67 (7.0)	80 (19.1)		147 (10.7)
Failed	87 (9.2)	19 (4.6)		106 (7.7)
Lost to follow-up	209 (22.0)	35 (8.4)		244 (17.8)

**Fig 1:**
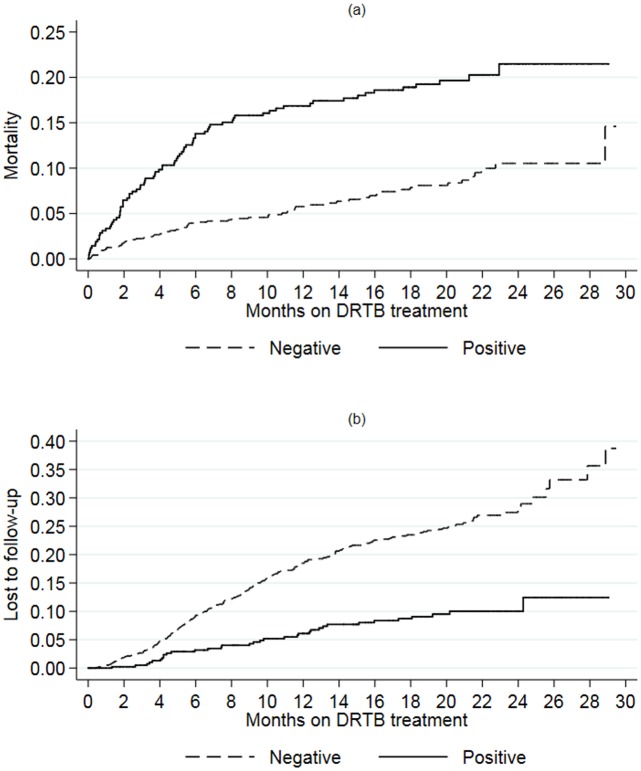
Kaplan-Meier estimates of mortality (a) and lost to follow-up (b) stratified by HIV status.

Overall, the median time to death was 5.5 months [IQR 2.0–13.9] among HIV-negative and 3.8 months [IQR 1.8–6.8] among HIV-positive patients. At 2 and 6 months the cumulative probability of death was 6.5% (95% CI 4.5–9.3%) vs 1.8% (95% CI 1.1–2.9%) and 13.8% (95% CI 10.8–17.5%) vs 3.9% (95% CI 2.9–5.4%) for HIV-positive and negative patients, respectively (Fig 1A). However, the lost to follow-up rate was much higher among HIV-negative patients (log-rank test: p<0.001, Fig 1B).

Culture conversion at 2 months could be assessed in 798/951 (83.9%) of HIV-negative patients and 282/418 (67.5%) of HIV-positive patients. It was not different between the 2 groups: 286/798 (35.8%) for HIV-negative and 90/282 (31.9%) for HIV-positive patients, p = 0.234.

### Risk factors for unsuccessful treatment outcomes for DRTB HIV-infected patients

A total of 134/418 (32.1%) DRTB HIV co-infected patients experienced an unsuccessful outcome, in a median time of 5.9 months [IQR 2.6–12.4] after DRTB treatment start.

Kaplan-Meier estimates of unsuccessful outcome rates reached 8.8% (95%CI 6.5–12.0%), 17.0% (95%CI 13.7–20.9%) and 23.3% (95%CI 19.6–27.7%) after 3, 6 and 12 months respectively (Fig A in S1 File).

Results of univariate and multivariate Cox model were presented in Table 3. The final multivariate model showed that age ≥ 35 years old (aHR = 1.53, 95%CI 1.05–2.24, p = 0.027) and not receiving ART were the only factors independently associated with an unsuccessful outcome. Indeed, patients who received ART before initiation of DRTB treatment had 67% decreased risk of experiencing unsuccessful outcome (aHR = 0.33, 95% CI 0.19–0.56, p<0.001); and patients who received ART after initiation of DRTB treatment had 73% decreased risk of experiencing unsuccessful outcome (aHR = 0.27, 95% CI 0.14–0.53, p<0.001) compared with those who never received ART (Table 3, Fig B in S1 File).

**Table 3:** Risk factors of unsuccessful (failure, death or lost to follow-up) DR-TB treatment outcome among patients HIV co-infected (N = 418).

	No. Unsuccessful/total	%	HR	95% CI	p-value	aHR	95% CI	p-value
**Sex**								
Female	61/223	27.3	Ref			Ref		
Male	73/195	37.4	0.97	(0.66–1.42)	0.864	0.85	(0.56–1.28)	0.442
**Age**								
< 35 years	62/236	26.3	Ref			Ref		
≥ 35 years	70/176	39.8	1.36	(0.96–1.95)	0.087	1.53	(1.05–2.24)	0.027
**Single**								
Yes	28/61	45.9	Ref					
No	35/89	39.3	0.80	(0.48–1.33)	0.391			
**Exprisoner**								
No	105/380	27.6	Ref					
Yes	29/38	76.3	1.22	(0.610–2.41)	0.573			
**Unemployed**								
No	13/35	37.1	Ref					
Yes	49/112	43.7	0.84	(0.44–1.62)	0.606			
**Contact of a MDR patient**								
No	130/406	32.0	Ref					
Yes	4/12	33.3	0.78	(0.28–2.18)	0.642			
**BMI**								
< 18.5	47/142	33.1	Ref			Ref		
≥ 18.5	65/244	26.6	0.76	(0.52–1.12)	0.170	0.69	(0.47–1.03)	0.071
**History of previous anti-TB treatment**								
New case	39/106	36.8	Ref			Ref		
Previously treated	89/294	30.3	0.76	(0.50–1.15)	0.190	0.72	(0.47–1.10)	0.131
**Any cavity**								
No	99/312	31.7	Ref					
Yes	35/106	33.0	0.73	(0.48–1.11)	0.136			
**Any other comorbidity**								
No	55/142	38.7	Ref					
Yes	79/276	28.6	0.48	(0.33–0.70)	<0.001			
**Smear result at t. initiation**								
Negative	37/116	31.9	Ref					
Positive	76/246	30.9	1.04	(0.69–1.57)	0.839			
**DST registration group**								
PDR	34/108	31.5	Ref			Ref		
MDR/XDR	76/211	36.0	0.87	(0.57–1.33)	0.519	0.90	(0.58–1.40)	0.639
**HIV treatment**								
No	60/101	59.4	Ref			Ref		
Before starting DRTB treatment	57/238	23.9	0.38	(0.23–0.64)	<0.001	0.33	(0.19–0.56)	<0.001
After starting DRTB treatment	17/79	21.5	0.33	(0.17–0.63)	0.001	0.27	(0.14–0.53)	<0.001
**Cotrimoxazol preventive treatment**								
No	6/9	66.6	Ref					
Yes	91/345	26.4	0.69	(0.22–2.14)	0.524			
**Project**								
Abkhazia	11/13	84.6	Ref			Ref		
Armenia	26/40	65.0	0.77	(0.37–1.59)	0.473	0.47	(0.22–1.02)	0.056
Colombia	1/1	100	1.57	(0.20–12.27)	0.669	1.09	(0.12–9.55)	0.934
Kenya	5/27	18.5	0.16	(0.05–0.47)	0.001	0.22	(0.06–0.90)	0.035
Kyrgyzstan	3/4	75.0	0.71	(0.19–2.57)	0.599	0.23	(0.05–0.95)	0.042
Swaziland	88/333	26.4	0.25	(0.13–0.47)	<0.001	0.60	(0.25–1.40)	0.237
**Year of starting treatment**								
≤ 2010	64/186	34.4	Ref			Ref		
> 2010	70/232	30.2	1.00	(0.71–1.41)	0.988	0.85	(0.56–1.27)	0.429

HR: Hazard ratio

Sensitivity analysis by excluding patients that were lost to follow-up (N = 35) showed similar results (ART before DRTB treatment vs. no ART, aHR = 0.28, 95%CI 0.16–0.50, p<0.001; ART after DRTB treatment vs. no ART, aHR = 0.17, 95%CI 0.08–0.37, p<0.001).

Although there is no difference in unsuccessful outcome rates between patients who interrupted DRTB treatment in first 3 months and those who didn’t (HR = 1.83, 95% CI 0.88–3.79, p = 0.103), a higher proportion of patients who did not receive ART interrupted treatment in the first 3 months compared to those who received ART (36.6% vs. 1.3%, p<0.001).

## Discussion

This study conducted among one of the largest cohorts of patients with DRTB shows that 30.5% of them were HIV-positive. The overall MDR-TB treatment success rate is 63.7% and is higher among HIV-positive patients, while no significant difference is shown according to HIV-status among PDR-TB patients. Older age and not receiving ART are the only factors associated with unfavorable treatment outcome among HIV-positive patients.

In our cohort, the prevalence of HIV was the same among PDR-TB patients (25.3%) and among MDR-TB patients (26.1%). Most HIV-positive patients came from Swaziland where the HIV prevalence is particularly high (27.7%, 95%CI 26.7–28.6 in 2014) [2]. HIV-positive patients were younger, more females and had less chest cavities as compared to HIV-negative patients as previously shown [11,12]. An incidence survey conducted in Swaziland has shown that the incidence of HIV was nearly twice as high in women as in men [13]. Patients included in this study are also more likely to present TB recurrence, which could potentially explain their higher exposure to previous first line anti-TB drugs [14]. Therefore, the socio-demographic characteristics of DR-TB HIV-positive patients in our cohort are very similar to what is generally observed among HIV-infected patients in sub-Saharan Africa. Treatment success rate in this cohort is higher than the 50% reported by WHO [1]. The higher success rate in HIV-positive patients are not consistent with what is usually observed in the TB drug susceptible population, where success rates are lower among HIV-positive due to higher death rate [15]. We also report a high case fatality rate among HIV-positive patients for both MDR and PDR-TB patients, likely to be attributable to HIV as already shown in previous studies [16–18]. Despite the higher proportion of rifampicin resistant PDR-TB among HIV-positive patients, the failure rate of PDR-TB treatment was similar between HIV-positive and negative patients. The absence of difference might be biased by the fact that there were more deaths occurring at an early stage in the group of HIV-positive patients. On the other hand, the failure rate of MDR-TB treatment was two folds lower in HIV-positive patients as compared to HIV-negative ones. Despite the absence of overall difference of resistance to 2^nd^ line anti-TB drugs between the two groups, there were more pre-XDR-TB patients with resistance to 2 injectables or fluoroquinolones and XDR-TB patients among HIV-negative patients (30.6%) compared to HIV-positive patients (21.0%). Patients with such resistance have an increased risk of failing MDR-TB treatment [19–22]. Other factors such as the higher proportion of lung cavitation, exposure to 2^nd^ line anti-TB drugs, and diabetes could also explain the higher failure rate of HIV-negative patients [23]. Lastly, no difference in culture conversion at 2 month were found between HIV-positive and HIV-negative patients, but this absence of difference could also be explained by the higher rate of early death in HIV-positive patients. Despite a higher rate of deaths among HIV-positive patients, the lower rate of failures combined with the lower rate of lost to follow-up can explain the overall higher rate of treatment success among HIV-positive patients compared to HIV-negative patients.

Several reasons could explain the difference of lost to follow-up rate between HIV-positive and HIV-negative patients in our cohort. Most HIV-positive patients are coming from Swaziland where the DR-TB program is decentralized improving access to treatment and treatment retention, compared to other programs in FSU countries where DR-TB treatment is more centralized [24]. Also, for patients receiving ART, one explanation could be that patients monitored at the same time for both HIV and TB treatments with scheduled clinic visit and ART refill might be less likely to be lost to follow-up [25]. The high rate of lost to follow-up among HIV-negative patients can be explained by the high proportion of resistance to injectable and fluoroquinolones resulting in poor treatment response and use of alternative 2^nd^ line drugs with very poor tolerability. Indeed, as shown in a previous study we carried out in Armenia, poor treatment tolerability and poor treatment response increased the risk of patients to stop their treatment [26,27]. Additional factors such as alcohol consumption that is very high among patients from FSU countries have been identified as risk factors for being lost to follow-up in other studies [28,29].

Among HIV-patients, those who did not received an ART before or at the start of DR-TB treatment were at higher risk of experiencing an unfavorable treatment outcome. This result, already shown, is not surprising since ART has clearly reduced morbidity and mortality of HIV-infected patients in high-, middle- and low-income countries [16,30–32]. WHO recommends ART initiation together with cotrimoxazole prophylaxis as soon as possible for DR-TB patients since 2011 [10]. One third of the patients from our cohort were enrolled before 2011, which could explain the delayed ART initiation. However, almost all co-infected patients received cotrimoxazole prophylaxis. Despite the acceptable overall lost to follow-up rate below 10% of HIV-positive DR-TB patients, there was a higher proportion of patients who interrupted treatment in the first 3 months among patients not on ART. Adherence is a well-known strong factor associated with DR-TB and HIV treatment success, and a lower adherence predict unsuccessful outcome [26,33,34]. Surprisingly, the profile of resistance at treatment start was not associated with DR-TB treatment outcomes among HIV-positive patients, while MDR-TB patients are expected to have worse outcomes than other DR-TB patients [21]. However, there is little data on treatment outcome of PDR-TB patients to illustrate this. Also the resistance profile of PDR-TB and MDR-TB was not so different because most PDR-TB patients were resistant to HE(S) and R(E)(S), and MDR-TB were mainly simple MDR without 2^nd^ line anti-TB drug resistance.

This study has several limitations. First, it was a retrospective study carried out on routinely collected data in programmatic conditions, hence to missing data. Indeed, DST results to second-line drugs were missing for an important proportion of MDR-TB patients. HIV-related data like CD4 cell count was missing, and this increases the difficulty to interpret treatment outcomes among HIV-positive patients. Adherence data to both DR-TB and HIV drugs were not available, thus we have used a proxy of adherence to DR-TB treatment represented by the presence of an interruption due to patient decision in the first three months of treatment. It could have been interesting to distinguish H(S) from the other PDR-TB profiles in the model to explore potential differences in successful rate, but this could not have been done due to the low sample size. Finally, most of HIV-infected patients came from Sub-Saharan Africa which could limit the generalizability of our results to other regions.

In conclusion, as shown for HIV-negative patients, success rate of DR-TB HIV-positive patients remains low and requires more effective DR-TB regimen using new drugs also suitable to HIV-infected patients on ART [35,36]. More data are needed on the safety and interaction of the bedaquiline and delamanid new anti-TB drugs with the antiretroviral drugs [37–41]. The study also confirms the importance of ART to improve treatment outcomes of DRTB HIV co-infected patients [14].

## Supporting information

S1 Fig Kaplan-Meier estimates of unsuccessful outcome: (a) overall; and (b) stratified by time of ART start. (EPS)

S1 Table Burden of HIV, TB and MDR-TB reported in 2016 by the World Health Organization. (DOCX)

## References

[1] World Health Organization. Global Tuberculosis Report [Internet]. 2016. http://apps.who.int/medicinedocs/documents/s23098en/s23098en.pdf

[2] UNAIDS. The Gap Report [Internet]. Geneva; 2014. http://www.unaids.org/sites/default/files/media_asset/UNAIDS_Gap_report_en.pdf

[3] Sanchez-Padilla E, Ardizzoni E, Sauvageot D, Ahoua L, Martin A, Varaine F, et al Multidrug- and isoniazid-resistant tuberculosis in three high HIV burden African regions. Int J Tuberc Lung Dis. 2013;17: 1036–42. 23827027 10.5588/ijtld.12.0842

[4] Sanchez-Padilla E, Dlamini T, Ascorra A, Rüsch-Gerdes S, Tefera ZD, Calain P, et al High prevalence of multidrug-resistant tuberculosis, Swaziland, 2009–2010. Emerg Infect Dis. 2012;18: 29–37. doi: 10.3201/eid1801.110850 22260950 10.3201/eid1801.110850 PMC3310109

[5] Mesfin YM, Hailemariam D, Biadgilign S, Biadglign S, Kibret KT. Association between HIV/AIDS and multi-drug resistance tuberculosis: a systematic review and meta-analysis. PLoS One. 2014;9: e82235 doi: 10.1371/journal.pone.0082235 24416139 10.1371/journal.pone.0082235 PMC3885391

[6] Dean AS, Zignol M, Falzon D, Getahun H, Floyd K. HIV and multidrug-resistant tuberculosis: overlapping epidemics. Eur Respir J. 2014;44: 251–4. doi: 10.1183/09031936.00205413 24525438 10.1183/09031936.00205413

[7] Isaakidis P, Casas EC, Das M, Tseretopoulou X, Ntzani EE, Ford N. Treatment outcomes for HIV and MDR-TB co-infected adults and children: systematic review and meta-analysis. Int J Tuberc Lung Dis. 2015;19: 969–978. doi: 10.5588/ijtld.15.0123 26162364 10.5588/ijtld.15.0123

[8] World Health Organization. Access to the WHO global TB database [Internet]. Geneva; 2016. http://www.who.int/tb/country/data/download/en/

[9] World Health Organization. Guidelines for the Programmatic Management of Drug-resistant Tuberculosis. 2008 Emergency Update. Geneva, Switzerland. [Internet]. 2008. http://whqlibdoc.who.int/publications/2008/9789241547581_eng.pdf

[10] World Health Organization. Guidelines for the Programmatic Management of Drug-resistant Tuberculosis. 2011 Update. Geneva, Switzerland. [Internet]. 2011. http://whqlibdoc.who.int/publications/2011/9789241501583_eng.pdf 23844450

[11] Getahun H, Sculier D, Sismanidis C, Grzemska M, Raviglione M. Prevention, Diagnosis, and Treatment of Tuberculosis in Children and Mothers: Evidence for Action for Maternal, Neonatal, and Child Health Services. J Infect Dis. 2012;205: S216–S227. doi: 10.1093/infdis/jis009 22448018 10.1093/infdis/jis009

[12] World Health Organization. Global AIDS update [Internet]. 2016. http://www.who.int/hiv/pub/arv/global-AIDS-update-2016_en.pdf?ua=1

[13] Justman J, Reed JB, Bicego G, Donnell D, Li K, Bock N, et al Swaziland HIV Incidence Measurement Survey (SHIMS): a prospective national cohort study. Lancet HIV. 2017;4: e83–e92. doi: 10.1016/S2352-3018(16)30190-4 27863998 10.1016/S2352-3018(16)30190-4 PMC5291824

[14] Harries AD, Lawn SD, Getahun H, Zachariah R, Havlir D V. HIV and tuberculosis—science and implementation to turn the tide and reduce deaths. J Int AIDS Soc. 2012;15: 17396 Available: http://www.ncbi.nlm.nih.gov/pubmed/22905358 22905358 10.7448/IAS.15.2.17396 PMC3499795

[15] Manda SO, Masenyetse LJ, Lancaster JL, van der Walt ML. Risk of Death among HIV Co-Infected Multidrug Resistant Tuberculosis Patients, Compared To Mortality in the General Population of South Africa. J AIDS Clin Res. 2013;Suppl 3: 7 doi: 10.4172/2155-6113.S3-007 24455448 10.4172/2155-6113.S3-007 PMC3894364

[16] Alobu I, Oshi SN, Oshi DC, Ukwaja KN. Risk factors of treatment default and death among tuberculosis patients in a resource-limited setting. Asian Pac J Trop Med. 2014;7: 977–84. doi: 10.1016/S1995-7645(14)60172-3 25479627 10.1016/S1995-7645(14)60172-3

[17] Ade S, Harries AD, Trébucq A, Ade G, Agodokpessi G, Adjonou C, et al National profile and treatment outcomes of patients with extrapulmonary tuberculosis in Bénin. PLoS One. 2014;9: e95603 doi: 10.1371/journal.pone.0095603 24755603 10.1371/journal.pone.0095603 PMC3995824

[18] Tweya H, Feldacker C, Phiri S, Ben-Smith A, Fenner L, Jahn A, et al Comparison of treatment outcomes of new smear-positive pulmonary tuberculosis patients by HIV and antiretroviral status in a TB/HIV clinic, Malawi. PLoS One. 2013;8: e56248 doi: 10.1371/journal.pone.0056248 23457534 10.1371/journal.pone.0056248 PMC3574145

[19] Falzon D, Gandhi N, Migliori GB, Sotgiu G, Cox HS, Holtz TH, et al Resistance to fluoroquinolones and second-line injectable drugs: impact on multidrug-resistant TB outcomes. Eur Respir J. 2013;42: 156–68. doi: 10.1183/09031936.00134712 23100499 10.1183/09031936.00134712 PMC4487776

[20] Kurbatova E V, Taylor A, Gammino VM, Bayona J, Becerra M, Danilovitz M, et al Predictors of poor outcomes among patients treated for multidrug-resistant tuberculosis at DOTS-plus projects. Tuberculosis (Edinb). 2012;92: 397–403. doi: 10.1016/j.tube.2012.06.003 22789497 10.1016/j.tube.2012.06.003 PMC4749016

[21] Bonnet M, Pardini M, Meacci F, Orrù G, Yesilkaya H, Jarosz T, et al Treatment of tuberculosis in a region with high drug resistance: outcomes, drug resistance amplification and re-infection. PLoS One. 2011;6: e23081 doi: 10.1371/journal.pone.0023081 21886778 10.1371/journal.pone.0023081 PMC3160294

[22] Bastos ML, Hussain H, Weyer K, Garcia-Garcia L, Leimane V, Leung CC, et al Treatment outcomes of patients with multidrug-resistant and extensively drug-resistant tuberculosis according to drug susceptibility testing to first- and second-line drugs: an individual patient data meta-analysis. Clin Infect Dis. 2014;59: 1364–74. doi: 10.1093/cid/ciu619 25097082 10.1093/cid/ciu619 PMC4296130

[23] Johnston JC, Shahidi NC, Sadatsafavi M, Fitzgerald JM. Treatment outcomes of multidrug-resistant tuberculosis: a systematic review and meta-analysis. PLoS One. 2009;4: e6914 doi: 10.1371/journal.pone.0006914 19742330 10.1371/journal.pone.0006914 PMC2735675

[24] Cox H, Ford N. Decentralisation of multidrug-resistant-tuberculosis care and management. Lancet Infect Dis. 2013;13: 644–646. doi: 10.1016/S1473-3099(13)70151-8 23743043 10.1016/S1473-3099(13)70151-8

[25] Shastri S, Naik B, Shet A, Rewari B, De Costa A. TB treatment outcomes among TB-HIV co-infections in Karnataka, India: how do these compare with non-HIV tuberculosis outcomes in the province? BMC Public Health. 2013;13: 838 doi: 10.1186/1471-2458-13-838 24025316 10.1186/1471-2458-13-838 PMC3850542

[26] Sanchez-Padilla E, Marquer C, Kalon S, Qayyum S, Hayrapetyan A, Varaine F, et al Reasons for defaulting from drug-resistant tuberculosis treatment in Armenia: a quantitative and qualitative study. Int J Tuberc Lung Dis. 2014;18: 160–7. doi: 10.5588/ijtld.13.0369 24429307 10.5588/ijtld.13.0369

[27] Post FA, Grint D, Werlinrud AM, Panteleev A, Riekstina V, Malashenkov EA, et al Multi-drug-resistant tuberculosis in HIV positive patients in Eastern Europe. J Infect. 2014;68: 259–63. doi: 10.1016/j.jinf.2013.09.034 24247067 10.1016/j.jinf.2013.09.034

[28] Kurbatova E V, Taylor A, Gammino VM, Bayona J, Becerra M, Danilovitz M, et al Predictors of poor outcomes among patients treated for multidrug-resistant tuberculosis at DOTS-plus projects. Tuberculosis (Edinb). 2012;92: 397–403. doi: 10.1016/j.tube.2012.06.003 22789497 10.1016/j.tube.2012.06.003 PMC4749016

[29] Sanchez-Padilla E, Marquer C, Kalon S, Qayyum S, Hayrapetyan A, Varaine F, et al Reasons for defaulting from drug-resistant tuberculosis treatment in Armenia: A quantitative and qualitative study. Int J Tuberc Lung Dis. 2014;18 doi: 10.5588/ijtld.13.0369 24429307 10.5588/ijtld.13.0369

[30] Padayatchi N, Abdool Karim SS, Naidoo K, Grobler A, Friedland G. Improved survival in multidrug-resistant tuberculosis patients receiving integrated tuberculosis and antiretroviral treatment in the SAPiT Trial. Int J Tuberc Lung Dis. 2014;18: 147–54. doi: 10.5588/ijtld.13.0627 24429305 10.5588/ijtld.13.0627 PMC4770013

[31] Palacios E, Franke M, Muñoz M, Hurtado R, Dallman R, Chalco K, et al HIV-positive patients treated for multidrug-resistant tuberculosis: clinical outcomes in the HAART era. Int J Tuberc Lung Dis. 2012;16: 348–54. doi: 10.5588/ijtld.11.0473 22640448 10.5588/ijtld.11.0473

[32] Dheda K, Shean K, Zumla A, Badri M, Streicher EM, Page-Shipp L, et al Early treatment outcomes and HIV status of patients with extensively drug-resistant tuberculosis in South Africa: a retrospective cohort study. Lancet. 2010;375: 1798–1807. doi: 10.1016/S0140-6736(10)60492-8 20488525 10.1016/S0140-6736(10)60492-8

[33] Bangsberg DR, Perry S, Charlebois ED, Clark RA, Roberston M, Zolopa AR, et al Non-adherence to highly active antiretroviral therapy predicts progression to AIDS. Aids. 2001;15: 1181–1183. 11416722 10.1097/00002030-200106150-00015

[34] Bastard M, Sanchez-Padilla E, Hewison C, Hayrapetyan A, Khurkhumal S, Varaine F, et al Effects of Treatment Interruption Patterns on Treatment Success Among Patients With Multidrug-Resistant Tuberculosis in Armenia and Abkhazia. J Infect Dis. 2014; doi: 10.1093/infdis/jiu551 25312040 10.1093/infdis/jiu551

[35] Diacon AH, Donald PR, Pym A, Grobusch M, Patientia RF, Mahanyele R, et al Randomized pilot trial of eight weeks of bedaquiline (TMC207) treatment for multidrug-resistant tuberculosis: long-term outcome, tolerability, and effect on emergence of drug resistance. Antimicrob Agents Chemother. 2012;56: 3271–6. doi: 10.1128/AAC.06126-11 22391540 10.1128/AAC.06126-11 PMC3370813

[36] Skripconoka V, Danilovits M, Pehme L, Tomson T, Skenders G, Kummik T, et al Delamanid improves outcomes and reduces mortality in multidrug-resistant tuberculosis. Eur Respir J. 2013;41: 1393–400. doi: 10.1183/09031936.00125812 23018916 10.1183/09031936.00125812 PMC3669462

[37] World Health Organization. Interim guidance on the use of bedaquiline to treat MDR-TB [Internet]. 2013. http://www.who.int/tb/challenges/mdr/bedaquiline/en/

[38] World Health Organization. The use of delamanid in the treatment of multidrug-resistant tuberculosis Interim policy guidance [Internet]. 2014. http://www.who.int/tb/publications/delamanid-in-mdr-tb-treatment/en/26110189

[39] Pandie M, Wiesner L, McIlleron H, Hughes J, Siwendu S, Conradie F, et al Drug—drug interactions between bedaquiline and the antiretrovirals lopinavir/ritonavir and nevirapine in HIV-infected patients with drug-resistant TB. J Antimicrob Chemother. 2016;71: 1037–1040. doi: 10.1093/jac/dkv447 26747099 10.1093/jac/dkv447

[40] Svensson EM, Dooley KE, Karlsson MO. Impact of Lopinavir-Ritonavir or Nevirapine on Bedaquiline Exposures and Potential Implications for Patients with Tuberculosis-HIV Coinfection. Antimicrob Agents Chemother. 2014;58: 6406–6412. doi: 10.1128/AAC.03246-14 25114140 10.1128/AAC.03246-14 PMC4249405

[41] Mallikaarjun S, Wells C, Petersen C, Paccaly A, Shoaf SE, Patil S, et al Delamanid Coadministered with Antiretroviral Drugs or Antituberculosis Drugs Shows No Clinically Relevant Drug-Drug Interactions in Healthy Subjects. Antimicrob Agents Chemother. 2016;60: 5976–5985. doi: 10.1128/AAC.00509-16 27458223 10.1128/AAC.00509-16 PMC5038266

