# Supplementary material for: Outcomes of HIV-infected versus HIV-non-infected patients treated for drug-resistance tuberculosis: Multicenter cohort study
Source: PLoS One. 2018 Mar 8;13(3):e0193491. doi: 10.1371/journal.pone.0193491 (PMC5843270; doi:10.1371/journal.pone.0193491)
Supplement: S1 Table — (DOCX) [file pone.0193491.s002.docx]

**S1 Table. Burden of HIV, TB and MDR-TB reported in 2016 by the World Health Organization.**

|  | **TB incidence**  **(/100,000 person-year)** | **HIV prevalence among adults (%)** | **HIV-TB co-infection incidence**  **(/100,000 person-year)** | **New TB cases with MDR-TB (%)** | **Previously treated TB cases with MDR-TB (%)** |
| --- | --- | --- | --- | --- | --- |
| **Armenia** | 41.0 (36.0 – 46.0) | 0.2 (0.2 – 0.3) | 3.7 (3.3 – 4.2) | 11.0 (8.0 – 14.0) | 47.0 (41.0 – 53.0) |
| **Georgia** | 99.0 (80.0 – 120.0) | 0.4 (0.3 – 0.5) | 6.4 (3.9 – 9.5) | 12.0 (11.0 – 14.0) | 33.0 (29.0 – 37.0) |
| **Colombia** | 31.0 (24.0 – 39.0) | 0.5 (0.4 – 0.5) | 4.4 (3.3 – 5.5) | 2.4 (1.4 – 3.4) | 14.0 (11.0 – 18.0) |
| **Kyrgyzstan** | 144.0 (120.0 – 170.0) | 0.2 (0.2 – 0.3) | 4.4 (2.8 – 6.3) | 32.0 (28.0 – 36.0) | 56.0 (53.0 – 59.0) |
| **Uzbekistan** | 79.0 (57.0 – 105.0) | 0.2 (0.1 – 0.2) | 3.9 (2.5 – 5.7) | 24.0 (18.0 – 30.0) | 63.0 (54.0 – 71.0) |
| **Swaziland** | 565.0 (366.0 – 807.0) | 28.8 (26.7 – 30.5) | 408.0 (261.0 – 586.0) | 8.0 (3.1 – 13.0) | 36.0 (31.0 – 42.0) |
| **Kenya** | 233.0 (189.0 – 281.0) | 5.9 (4.9 – 7.0) | 78.0 (63.0 -94.0) | 1.3 (0.7 – 1.9) | 9.4 (8.7 – 10.0) |
